# Antioxidant and Antimicrobial Activity of Microalgae of the Filinskaya Bay (Baltic Sea)

**DOI:** 10.3390/plants11172264

**Published:** 2022-08-31

**Authors:** Alexandra Shevelyuhina, Olga Babich, Stanislav Sukhikh, Svetlana Ivanova, Egor Kashirskih, Vitaliy Smirnov, Philippe Michaud, Evgeny Chupakhin

**Affiliations:** 1 Institute of Living Systems, Immanuel Kant Baltic Federal University, A. Nevskogo Street 14, Kaliningrad 236016, Russia; 2 Natural Nutraceutical Biotesting Laboratory, Kemerovo State University, Krasnaya Street 6, Kemerovo 650043, Russia; 3 Department of General Mathematics and Informatics, Kemerovo State University, Krasnaya Street 6, Kemerovo 650043, Russia; 4 Rusextract, Tereshkovoy Street 51, Kemerovo 650070, Russia; 5 Sodrugestvo Group, Gagarina Street 65, Svetly, Kaliningrad 238340, Russia; 6 Institut Pascal, Université Clermont Auvergne, CNRS, Clermont Auvergne INP, 63000 Clermont-Ferrand, France

**Keywords:** microalgae, *S. intermedius*, *S. obliquus*, proteins, lipids, reducing sugars, antioxidant and antimicrobial activity, inhibition zone

## Abstract

Microalgae are rich in proteins, carbohydrates, lipids, polyunsaturated fatty acids, vitamins, pigments, enzymes, and other biologically active substances. This research aimed to study the composition and antioxidant and antimicrobial activity of proteins, lipids, and carbohydrates of microalgae found in the Filinskaya Bay of the Kaliningrad region. The chemical composition of *Scenedesmus intermedius* and *Scenedesmus obliquus* microalgae biomass was studied. Ultrasound was used to isolate valuable components of microalgae. It was found that microalgae are rich in protein and contain lipids and reducing sugars. To confirm the accuracy of the determination, the protein content was measured using two methods (Kjeldahl and Bradford). Protein content in *S. intermedius* and *S. obliquus* microalgae samples did not differ significantly when measured using different methods. Protein extraction by the Kjeldahl method was found to be 63.27% for *S. intermedius* microalgae samples and 60.11% for *S. obliquus* microalgae samples. Protein content in *S. intermedius* samples was 63.46%, compared to 60.07% in *S. obliquus* samples, as determined by the Bradford method. Lipids were 8.0–8.2 times less abundant than protein in both types of microalgae samples. It was determined that the content of reducing sugars in the samples of the studied microalgae was 5.9 times less than the protein content. The presence of biological activity (antioxidant) in proteins and lipids obtained from biomass samples of the studied microscopic algae was established, which opens up some possibilities for their application in the food, chemical, and pharmaceutical industries (as enzymes, hormones, vitamins, growth substances, antibiotics, and other biologically active compounds).

## 1. Introduction

Microalgae include a wide range of autotrophic organisms that grow through photosynthesis in the same way as plants. Microalgae classification is constantly being revised in response to new genetic data. Nevertheless, two main groups of microalgae have been distinguished: prokaryotic and eukaryotic [1]. Microalgae samples have recently piqued the interest of researchers due to their properties and characteristics (the ability to synthesize various biologically active substances, rapid biomass growth, and the possibility of external regulation of their biochemical composition by cultivation conditions) [1,2,3]. Unlike heterotrophic microorganisms that need various organic compounds for growth, unicellular photosynthetic organisms produce biomass from fully oxidized inorganic substances and mineral elements due to light energy being converted during photosynthesis. Furthermore, microalgae biomass production technologies do not pollute the environment, use carbon dioxide while releasing oxygen, use a small amount of water, and may occupy land resources unsuitable for crop cultivation [1].

Microalgae are currently used in two main areas: biomass generation for direct use as a biologically active additive (BAA) and subsequent isolation of biologically active substances (BAS) [4,5,6]. These compounds are in demand in medicine, cosmetology, food industry, fish farming, energy, agriculture, feed, and functional food production. Microalgae-derived biologically active substances have antioxidant, immunostimulatory, antibacterial, antiviral, antitumor, antihypertensive, regenerative, and neuroprotective properties [2,7].

The introduction of microalgae as a new crop in modern agriculture can improve its efficiency and increase the production of food, environmentally friendly chemicals, and modern biofuels in a more sustainable way. In addition, microalgae can be cultivated through environmentally friendly processes based on the use of anthropogenic emissions such as carbon dioxide and wastewater. However, the introduction of a new biomass, such as microalgae, to the market is a long-term process that requires the improvement of the technological process (improvement of strains [8], development of new process organization schemes [9], large-scale investments [10], etc.).

Only a few microalgae species (*Arthrospira*, *Chlorella*, *Dunaliella*, *Aphanizomenon*, and *Nostoc*) have been approved for human consumption to date [11,12,13,14,15]. These microalgae are a promising object for large-scale cultivation due to the high content of biologically active substances and the relatively cheap production process. Other microalgae species, such as *Chlamydomonas* sp., *Chlorococcum* sp., *Scenedesmus* sp., *Tetraselmis chuii*, and *Nanochloropsis* sp., have been shown to be useful in aquaculture, feed, fertilizer, and cosmetics but do not have Generally Recognized as Safe (GRAS) designation yet [5,16,17,18,19,20,21,22].

The main species of microalgae and cyanobacteria inhibiting the Baltic Sea are presented in Table 1.

Information on the spread of microalgae species such as *Scenedesmus intermedius* and *Scenedesmus obliquus* in the Baltic Sea (Table 1) is insufficient; further research is needed to determine their presence, growth potential, and content of biologically active substances, including in Filinskaya Bay.

The microalgae *S. intermedius* and *S. obliquus* are axenoic marine microorganisms consisting of a single eukaryotic cell, and the presence of bacterial cells was not detected during the identification of microalgae. The genus *Scenedesmus* is primarily found along the Baltic Sea coast, specifically in Filinskaya Bay. The microalgae *S. intermedius* and *S. obliquus* are quite rare. It is these microalgae that can serve as valuable food and feed raw materials, the industrial use of which requires an assessment and forecast of possible intake volumes [23]. For year-round production of proteins, lipids, and carbohydrates of constant composition and properties from microalgae, *S. intermedius* and *S. obliquus* must be cultivated under rational conditions with the selection of process parameters (temperature, cultivation duration, degree of aeration) to obtain the greatest amount of useful biomass, the achievement of which in natural conditions is not possible.

*S. intermedius* and *S. obliquus* microalgae strains show promise for producing enhanced biofuels [24], as well as rapid growth and high protein production when grown on fertilizer-based media [25]. However, the potential of rare microalgae *S. intermedius* and *S. obliquus* of the Filinskaya Bay is poorly understood and requires the constant availability of their biomass grown under the same rational conditions [23].

The search for new or understudied microalgae strains can open up new opportunities for their use by broadening the scope of their industrial application [24,25,26]. The true potential of microalgae has not been fully assessed due to their great diversity, high metabolic flexibility, and diverse cultivation conditions [27,28,29,30]. Innovative developments to optimize microalgae production will make their use economically feasible and in demand in the Baltic region due to the significant amount of their biomass in the coastal zone of the Baltic Sea. This research aimed to study the composition and antioxidant and antimicrobial activity of proteins, lipids, and carbohydrates of microalgae sampled in the Filinskaya Bay (Kaliningrad region, Russia). As a scientific novelty, for the first time, microalgae were sampled from the Filinskaya Bay, the coast of the Baltic Sea, the Kaliningrad region, and the collected samples were identified (*S. intermedius* and *S. obliquus*)*;* for the first time, the protein, lipid, and carbohydrate composition was evaluated; antioxidant and antimicrobial properties of *S. intermedius* and *S. obliquus* extracts were studied [31].

## 2. Results

### 2.1. Microalgae Cultivation

**Figure 1:**
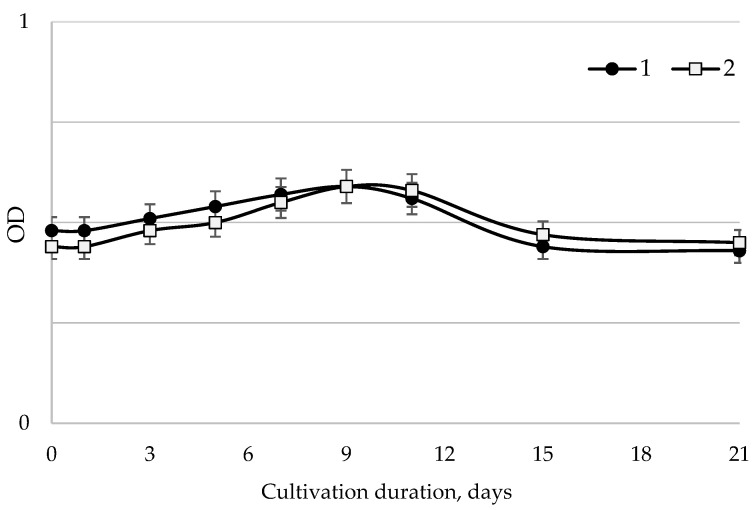
The dependence of the optical density of the Filinskaya Bay microalgae suspension on cultivation time: 1—*S. intermedius*; 2—*S. obliquus*.

The dynamics of the optical density of microalgae suspension were used to investigate cultivation patterns (Figure 1).

Optical density or turbidity is an indirect method for determining cell concentration. Optical density is the logarithmic ratio of scattered light to light transmitted through a bacterial mixture. It is proportionally related to the concentration of bacteria (as well as microalgae) when the optical density is changing from 0.1 to 0.8. This rapid method of reading biomass samples and obtaining an exact cell concentration was made possible by the standard absorbance-to-dry-cell-weight (DCW) curve.

Light is one of the main parameters affecting the formation and growth of microalgae. It provides energy to support metabolism, but when excessive, it can lead to oxidative stress and growth inhibition. In the process of cultivation, *S. obliquus* and *S. intermedius* showed the maximum growth rate at 150 mcM photons m^−2^ s^−1^. Below this value, light was limiting for growth, and under more intense illumination, the effects of photosaturation were observed, although the cells still showed the ability to reproduce.

Judging by the biochemical composition, light only affected the pigment content, while the content of carbohydrates, lipids, and proteins remained stable. Considering that microalgae cells were subjected to light–dark cycles due to mixing during cultivation, microalgae were also grown under pulsed illumination (5, 10, and 15 Hz). At a low light intensity in the range from 10 to 150 mcM m^−2^ s^−1^, the specific growth rate and concentration of microalgae cells increased. An increase in light intensity did not lead to an increase in the growth rate, nor to an increase in the final concentration of cells, which indicates that the saturation point of photosynthesis has been reached.

These results differ from those reported by Liu et al. [32], who observed the maximum growth rate at higher intensities (250–400 mcM photons m^−2^ s^−1^) for *Scenedesmus* sp. However, this can be explained by the different depth of cultivation (about 4 cm [32]), which probably caused some self-darkening effect. If this is the case, then the average exposure in culture is much lower than the incident light. It is also worth mentioning that during cultivation, other types of microalgae also showed photosaturation at the illumination of more than 150 mcM m^−2^ s^−1^ photons [33]. Above 150 mcM m^−2^ s^−1^, the final dry biomass of microalgae increased with the intensity of light even if the number of cells did not change, which indicated that under these conditions, the cells increased their weight. In fact, despite a decrease in the concentration of cells from 40 to 30%, at a high light intensity (350 and 1000 mcM m^−2^ s^−1^), there is an increase in dry weight from about 25 to 50% compared to 150 mcM m^−2^ s^−1^.

When analyzing the concentration of pigment, the cells exposed to different light intensities showed a decrease in the content of chlorophyll in the cell and an increase in the content of carotenoids. Thus, like other photosynthetic organisms, *S. obliquus* and *S. intermedius* demonstrated an acclimatization reaction due to a decrease in chlorophyll content and an accumulation of carotenoids with antioxidant activity and the ability to absorb less light.

The biochemical composition of total proteins, lipids, and carbohydrates was analyzed for all illumination conditions except 10 mcM m^−2^ s^−1^, where low biomass yields limited measurements. The content of proteins, carbohydrates, and lipids in *S. obliquus* and *S. intermedius* cells did not show significant changes at different light intensities. In particular, the lipid content is the same in all conditions and is about 40% on dry basis. Unlike other algae species, such as *Nannochloropsis* [34,35], excess light did not cause overproduction of lipids. It is assumed that these reactions are not characteristic of all microalgae but depend on the species [36,37]. These data are consistent with the data from Breuer et al. [38], who reported that the maximum content of biologically active substances in *Scenedesmus* does not depend on the light intensity, while it strongly depends on pH and temperature.

As mentioned earlier, microalgae during cultivation can move from completely open areas into darkness. To study the effect of illumination on microalgae in photobioreactors on an industrial scale, it is very important to understand how these conditions affect the growth of algae. For this reason, *S. obliquus* and *S. intermedius* cells were grown with the dark–light cycle changing when exposed to light of different frequencies. Previously, the effect of the high-frequency light–dark cycle on *S. obliquus* was studied by Grobbelaarl et al. [39], but it remains unclear how these cycles can affect the productivity of the biomass of this species. For this reason, the specific growth rate and the final concentration of the biomass are reported depending on the different frequencies of the light–dark cycles. It was found that the different duration of the light and dark phases always provided the total amount of energy corresponding to the optimal intensity of 150 mcM m^−2^ s^−1^. In contrast to what was observed for *N. salina* [32], *S. obliquus* and *S. intermedius* demonstrated a slow-down in growth under pulsed illumination compared to the exposure to the same number of photons continuously in all cases. *S. obliquus* and *S. intermedius* grow better at a frequency of 10 Hz than in other conditions.

Xue et al. [40] reported an increase in growth at 10 Hz compared to 0.1 Hz, but they did not make a comparison with continuous illumination. It was found that even when using a frequency of 10 Hz, the growth of *S. obliquus* and *S. intermedius* was higher than in other cases. It was noted that pulsed light was even more harmful than the same amount of intense light (1000 mcM m^−2^ s^−1^) under the condition of continuity. The presented experimental results showed that the green microalgae *S. obliquus* and *S. intermedius* exhibit a significant growth rate even at intensities exceeding the optimal one. These results confirm the observation of Masojidek et al. [41], who reported a low sensitivity of *Scenedesmus* to photoinhibition and a higher ability to adapt to intense illumination due to an effective extinguishing mechanism and high photosynthetic ability. This ability to use suboptimal light makes the use of *S. obliquus* and *S. intermedius* particularly suitable for large-scale outdoor production in which lighting conditions are not controlled.

### 2.2. Microalgae Species Identification

The studied samples are characterized by spherical microscopic shapes with a continuous, solid, smooth surface. The size of microalgae was 5–20 μm, and it was found that they are able to form colonies reaching 1.5 cm. The simplicity of the external form of the microalgae made it difficult to classify according to morphological features. Because of this, the 18S ribosomal RNA gene was sequenced to obtain additional information on microalgae species.

Sequencing of the 18S rRNA gene (Appendix A and Appendix B) showed that the studied samples belong to *Scenedesmus intermedius* and *Scenedesmus obliquus*.

### 2.3. Study of Microalgae Component Composition

The content of proteins, lipids, and reducing sugars in microalgae samples without pretreatment and after sonication is presented in Table 2.

### 2.4. Determination of Antioxidant Activity of Alcohol Extracts of Microalgae

Table 3 shows antioxidant activity indicators for the microalgae alcohol extracts of proteins and acetone extracts of lipids.

Indicators of antimicrobial activity of microalgae *S. intermedius* and *S. obliquus* are presented in Table 4.

## 3. Discussion

According to the standard curve of the ratio of optical density to the dried cell weight, it can be concluded that for the first 10 days of cultivation, the value of optical density increased due to an increase in the number of microalgae. Further, the growth rate of microalgae decreased, and the values of the optical density of the last 5 days had no significant differences (Figure 1).

The traditional methods for isolating proteins, lipids, and carbohydrates from microalgae are cold pressing (soft pressing under normal conditions), hot pressing (pressing at 180 ± 20 °C, with filtration), water–steam extraction (steam is passed through the raw material, with which valuable substances are subsequently removed by decantation), water–alcohol extraction (washing of raw materials with a 60–80% alcohol solution, followed by drying), oil extraction (dissolution in heated vegetable oil), and solvent extraction (isolation of alcohol, ether, hydrocarbon solvents of waxes and oils, which are then separated by other solvents and evaporation) [42]. These methods have a number of disadvantages: they can only be used for soft, easily pressed plant tissues, heating removes some of the valuable properties of microalgae, the processes are labor- and energy-intensive, some of the valuable substances insoluble in alcohol remain in the cake, there is a need for recycled alcohol, instability of the properties of extracts, and destruction of a number of valuable components [42].

Ultrasound was found to be an environmentally friendly alternative to traditional methods for extracting microalgae components. The effect of ultrasound can be attributed to bubble cavitation, which contributes to the destruction of biological matrices and the subsequent release of proteins. In the case of pre-treatment in an ultrasonic bath, the cavitation process and the destruction of the microalgae matrix occurred more intensively; therefore, the extraction efficiency increased (Table 2).

According to the findings, protein extraction from microalgae *S. intermedius* samples using the Kjeldahl method was 63.27%, whereas protein extraction from microalgae *S. obliquus* samples was 60.11%. *S. intermedius* samples contained 63.46%, while *S. obliquus* samples had 60.07%, according to the Bradford method. Lipids are 8.0–8.2 times less abundant than protein in both types of microalgae samples. According to the Kjeldahl method, the reducing sugar content in the microalgae *S. intermedius* samples was 6.6 times less than the protein content; in the microalgae *S. obliquus* samples, the reducing sugar content was 5.9 times less than the protein content.

When compared to data from other studies [42,43,44], it was found that *Scenedesmus* sp. extracts obtained from the *Scenedesmus* sp. biomass samples grown in the laboratory contained up to 44% proteins, up to 6% lipids, and up to 7% carbohydrates.

The study showed that when proteins were extracted from *S. obliquus* samples with 0.1 mol L^−1^ NaOH solution at 95 °C for 30 min, the protein content was 58.7 ± 0.5% (by the Bradford method). Carbohydrates were extracted with a 4 mol/L sulfuric acid solution at 90 °C for 90 min, and the concentration of total neutral carbohydrates was determined using the phenol sulfuric acid method. The carbohydrate content was 26.2 ± 0.3%.

The total lipid content in the suspension of *S. obliquus* was determined using a gravimetric method based on the Schmid–Bondzynski–Ratzlaff principle, which consists in the hydrolysis of microalgae samples with hydrochloric acid followed by lipid extraction with ethanol, diethyl ether, and petroleum ether [44]. The lipid content in the studied samples was 21.0 ± 1.0%.

In [45], protein extraction was carried out by alkaline hydrolysis followed by precipitation with trichloroacetic acid. The protein content in this extraction method was 53.2%. The content of lipids in samples of *S. obliquus* microalgae after extraction without sonification, as in our experiment, was 12.5% and 22.0% carbohydrates.

Table 5 presents an analysis of the results on the content of proteins, lipids, and carbohydrates in *S. typhimurium* and *S. obliquus* microalgae obtained in this study with the results of studies by other scientists.

In biological systems, reactive oxygen and/or nitrogen species damage DNA molecules and lead to the oxidation of lipids and proteins in cells. The introduction of plant antioxidant components into the daily diet helps to reduce the intensity of free-radical processes in the cell and thereby protects it from oxidative damage. The mechanism of action of antioxidants is related to the fact that they interact with active radicals to form low-active radicals or destroy hydroperoxides, donating electrons without becoming aggressive. The antioxidant is consumed during the reaction. In this regard, in addition to direct-acting antioxidants, synergistic substances, which do not have antioxidant properties but enhance the antioxidant effect by acting as hydrogen donors, are required.

It was found that proteins and lipids of the studied microalgae samples have an antioxidant effect. It was discovered that the antioxidant activity of protein concentrate samples is significant and varies from 335.32 mg/mL (IC50) to 33016 mg/mL (IC50) for *S. intermedius* and *S. obliquus*, respectively. Moreover, the results presented in Table 4 indicate that microalgae lipids have insignificant antioxidant activity: 0.0026 mg/mL (IC50) for *S. intermedius* and 0.0013 mg/mL (IC50) for *S. obliquus*. The results obtained are 3.5 times less than the antioxidant activity of the control (ascorbic acid) for *S. intermedius* and 6.6 times for samples of microalgae *S. obliquus*. The results allowed for the conclusion that the antioxidant activity of reducing sugars was absent in both types of microalgae samples.

Table 6 presents the results of studying the antioxidant activity in *S. typhimurium* and *S. obliquus* microalgae obtained in various studies

The results of our research are supported by the findings of other studies. The study determined the activity of the *S. obliquus* extract in scavenging free radicals (antioxidant activity). It was found that the antioxidant activity of *S. obliquus* lipids was 0.00125 mg/mL, which is in good agreement with our data (0.0026 mg/mL for *S. intermedius* and 0.0013 mg/mL for *S. obliquus*). It was found that proteins of the *S. intermedius* microalgae exhibited antibiotic activity against *P. vulgaris* (inhibition zone diameter was 13.0 ± 0.4 mm), *S. aureus* (inhibition zone diameter was 8.0 ± 0.2 mm), and *C. albicans* (inhibition zone diameter was 2.0 ± 0.1 mm), and the proteins of *S. obliquus* microalgae were active against *P. vulgaris* (inhibition zone diameter was 6.0 ± 0.2 mm) and *C. albicans* (inhibition zone diameter was 3.0 ± 0.1 mm) [46].

Analysis of the antibacterial activity of microalgae lipids (*S. intermedius* and *S. obliquus*) indicated that both *S. intermedius* and *S. obliquus* lipids had the highest activity against the studied opportunistic and pathogenic microorganisms. Thus, *S. intermedius* lipids inhibited the growth and development of E. coli (inhibition zone diameter was 10.0 ± 0.3 mm) and *C. albicans* (inhibition zone diameter was 11.0 ± 0.3 mm). The studied lipids of *S. obliquus* samples were able to inhibit the vital functions of pathogenic and opportunistic microorganisms such as *S. aureus* (inhibition zone diameter was 12.0 ± 0.3 mm), *B. subtilis* (inhibition zone diameter was 16.0 ± 0.5 mm), and *C. albicans* (inhibition zone diameter was 10.0 ± 0.3 mm). Lipids of both microalgae species exhibited antibacterial activity against *P. vulgaris*; however, the lysis zone had a smaller diameter (4.0 ± 0.3 mm for *S. intermedius*, 3.0 ± 0.3 mm for *S. obliquus*).

Reducing sugars of *S. intermedius* and *S. obliquus* microalgae samples exhibited antibacterial activity against *P. vulgaris*, *E. coli*, *B. subtilis*, *S. aureus*, and *C. albicans*. For example, sugars from *S. intermedius* samples inhibited the growth of *P. vulgaris* (inhibition zone diameter 16.0 ± 0.5 mm), *E. coli* (inhibition zone diameter 18.0 ± 0.5 mm), *B. subtilis* (inhibition zone diameter 16.0 ± 0.5 mm), *S. aureus* (inhibition zone diameter 12.0 ± 0.6 mm), *C. albicans* (inhibition zone diameter 18.0 ± 0.5 mm); *S. obliquus* lipids inhibit the growth of *P. vulgaris* (inhibition zone diameter 16.0 ± 0.5 mm), *E. coli* (inhibition zone diameter 12.0 ± 0.3 mm), *B. subtilis* (inhibition zone diameter 14.0 ± 0.4 mm), *S. aureus* (inhibition zone diameter 19.0 ± 0.6 mm), and *C. albicans* (inhibition zone diameter 17.0 ± 0.5 mm). Analysis of the results presented in Table 5 showed that proteins, lipids, and reducing sugars of the studied samples of *S. intermedius* and *S. obliquus* microalgae had antibacterial properties against the studied test strains. The results obtained in our work are similar to those of other researchers [47].

Table 7 presents the results of studying the antimicrobial activity in *S. typhimurium* and *S. obliquus* microalgae samples obtained in various studies.

The study [47] tested the antimicrobial activity of *S. obliquus* microalgae extracts on three foodborne pathogens: *S. aureus*, *E. coli*, and *S. typhimurium*. The results of the disk diffusion test showed that the diameter of the zone of inhibition of *S. obliquus* extracts was 12.5 mm for *E. coli* and *S. typhimurium* and more than 12.5 mm for *S. aureus*. The study compared antibacterial activity of *S. obliquus* reducing sugars against *S. aureus*, *E. coli*, *P. vulgaris*, and *C. albicans* with vancomycin. The zone of inhibition with respect to *S. aureus* was the largest, measuring 20 mm in diameter (in our study, the inhibition zone diameter was 19 mm) [48].

## 4. Materials and Methods

### 4.1. Microalgae Sampling

Microalgae were sampled from peat and water in the Zelenograd region on the coast of the Baltic Sea (54°57′22.44″ and 20°28′28.93″, Kaliningrad region, Russia) in the period from July to September 2021.

Samples were taken using a plankton net [49]. The plankton net consisted of a brass ring and a conical bag sewn to it from a mill silk or nylon mesh net or another type. A vessel was tightly attached to the narrow outlet, which had an outlet tube closed with a tap or Mohr’s clamp. When collecting plankton from the surface layers of water, the plankton net was lowered into the water so that the upper opening of the net was 5–10 cm above its surface. The water was scooped from the surface layer (up to 15–20 cm deep) with a liter mug and poured into the net, thus filtering 50–100 L of water. Plankton samples were taken from the boat. At the same time, a plankton net was pulled on a thin rope behind a moving boat for 5–10 min.

After completing the plankton collection, the plankton net was rinsed to wash off the algae that was stuck on the inner surface of the net. The plankton sample concentrated in this way, which was in the vessel of the plankton net, was poured through the outlet tube into a clean jar or bottle prepared in advance.

The microalgae were transferred to the aquarium with sea water and then to flat-bottomed 250 mL flasks with a nutrient medium. The composition of the nutrient medium was 1 L of distilled water, ammonium nitrate 200 mg, sodium carbonate 500 mg, manganese chloride 0.05 g, anhydrous calcium (chloride) 100 mg, guanosine 200 mg, liquid fertilizer for plants, in which the ratio of elements N:P:K was 13:15:18, the concentration of the solutions was 0.1%, and the bioavailability of fertilizers was 75%. The nutrient media described above were used as a control (base case) to assess how different medium components affected microalgae development. The conditions for cultivating microalgae biomass in a standard medium in flasks (temperature and light conditions: 25 °C, 12 µmol/s, pH 6.2–6.4) were selected.

Next, the microalgae biomass was grown in a Sartorius BIOSTAT A bioreactor (Sartoros, Moscow, Russia) at T = 24 °C, pH = 6.6, stirring 300 rpm, continuous illumination 12 µmol m^−2^ s^−1^. The total incubation time was 3 weeks.

All reagents and nutrient media were purchased from Diaem (Moscow, Russia).

### 4.2. Analysis of Microalgae Biomass by Spectrophotometry

The relationship between optical density (OD) and dried cell weight (DCW) at 550 and 690 nm in g/L was experimentally determined from a 10-point curve. Optical density was measured at 550 and 690 nm using a Shimadzu UV-3600 spectrophotometer (Shimadzu, Kyoto, Japan). Samples were taken from a Sartorius BIOSTAT A bioreactor (Sartoros, Moscow, Russia).

Five milliliter samples were centrifuged for 10 min at 6000 g. The supernatant was removed, and the precipitate was resuspended in 5 mL of distilled water. The optical density was read within 5 min relative to a blank sample freshly prepared at the time of sampling. The samples were diluted as needed (to read the values of optical density in the range from 0.2 to 0.7).

### 4.3. Determination of the Dry Mass of Microalgae

The following procedure was followed to determine the dry cell weight (DCW) of microalgae: taking a 5 mL sample of the culture liquid from the bioreactor, centrifugation at 5000 rpm for 10 min, draining the supernatant and washing of sediment with distilled water from the cylinder (5 mL) with a glass rod (the remaining 5 mL of water was used to wash off the microalgae on the rod), one more centrifugation for 10 min at 5000 rpm, and transfer to weighing bottles with a known mass and then drying at 105 °C (the bottle was placed in an oven) to constant weight.

Based on the obtained data, optical density against time diagram was plotted.

### 4.4. Microalgae Species Identification

Identification was carried out by morphological comparison according to a photo from the guide of cultivated microalgae using the collection of microalgae and cyanobacteria of the K.A. Timiryazev Institute of Plant Physiology of the Russian Academy of Sciences (IPPAS IPP RAS). Morphology of microalgae was determined at 40× magnification using a Micros binocular microscope (Micros, Vienna, Austria).

Microalgae were also identified based on the polymerase chain reaction (PCR). Genetic identification of the obtained cultures of microalgae and cyanobacteria was carried out by sequencing 18s rRNA gene fragments using the Sanger method. PCR mixtures were prepared in a volume of 50 uL and included: 0.2 uM forward and reverse primers, master mix qPCRmix-HS SYBR (Evrogen, Moscow, Russia,), and ~10–30 ng of DNA for one reaction. Fragment amplification was carried out in a CFX-96 thermal cycler (BioRad, Berkeley, CA, USA) according to the following program: 95 °C—3 min ⇒ 28 cycles (95 °C—30 s; 55–60 °C—30 s; 72 °C—30 s) ⇒ 72 °C—3 min. The PCR products were sized and purified by preparative gel electrophoresis using the Cleanup Mini kit (Evrogen, Moscow, Russia). Two-way sequencing in the directions (5′-3′) and (3′-5′) was performed on a 3500 genetic analyzer (Applied Biosystems, Wakefield, MA, USA).

Using partial sequences of the gene encoding small subunits of ribosomes, 18S rRNA was determined. Primers were purchased from Evrogen (Moscow, Russia).

### 4.5. Determination of Antioxidant Activity of Alcohol Extracts of Microalgae

For the extraction of antioxidants (extracts of proteins, lipids, or sugars), 0.1 g of crushed dry algae was mixed with 10 mL of 96% (*v*/*v*) ethanol (diluted with distilled water), filtered through a paper filter. The filtrate was collected and used as the crude extract.

The activity of the extract is scavenging 2-2-diphenyl-1-picrylhydrazyl (DPPH) free radicals [49]. A 0.2 mM solution of DPPH in ethanol was prepared and added to 2 mL of the extracted samples at various concentrations (0.5, 1.0, and 2.0 mg/mL). After a 30 min incubation, the optical density was measured on a UV-3600 spectrophotometer (Shimadzu, Kyoto, Japan) at a wavelength of 517 nm. Ascorbic acid at various concentrations (from 0.00125 to 0.008 mg/mL) was used as a control for antioxidant activity. The percentage of DPPH inhibition was calculated using the equation:

absorption effect (%) = (1 − absorbance sample / absorbance control) × 100%(1)

A plot of absorption activity versus sample concentration was created and the IC50, or effective concentration at which 50% of the DPPH radicals were absorbed, was determined using linear regression.

### 4.6. Determination of the Microalgae Protein Content Using the Bradford Method

Protein was extracted following Barbarino and Lourenço [50]. An amount of 50 mg of dried microalgae biomass was mixed with 4 mL of distilled water and incubated at 4 °C for 12 h. Then, the algae mixture was ground in a mortar with a pestle for 5 min and incubated at 4 °C for another 1 h. After grinding, the mixture was centrifuged (at 4 °C, 15,000× *g*) for 20 min. The supernatant was collected, and the algae precipitate was re-extracted with 1 mL of 0.1 n NaOH with 0.5% β-mercaptoethanol (*v*/*v*). The mixture of algae extract and NaOH solution was kept at room temperature for 1 h and periodically shaken by hand. Then, the mixture was centrifuged for 20 min at 21 °C, 15,000× *g*. Then, the protein content was determined using the Bradford method [51].

The antioxidant activity of the isolated proteins in scavenging 2-2-diphenyl-1-picrylhydrazyl (DPPH) free radicals was determined as previously described (Section 4.5).

### 4.7. Determination of the Microalgae Protein Content Using the Kjeldahl Method

The crude protein content in the sample on absolute dry matter (a.d.m.) was determined using the Kjeldahl method [52]. The method is based on the mineralization of organic matter with sulfuric acid in the presence of a catalyst, which results in the formation of ammonium sulfate, the destruction of ammonium sulfate with alkali, which results in the release of ammonia, and the stripping of ammonia with water vapor into a sulfuric or boric acid solution, followed by titration. Then, the mass fraction of nitrogen and crude protein content was calculated (by multiplying by a factor of 6.25) [52].

### 4.8. Determination of the Microalgae Lipid Content

Total lipids were extracted using a mixture of chloroform and methanol (2:1, *v*/*v*) according to the Folch method [53] with cell disruption by sonication. An amount of 20 mL of solvent was added to 1 g of dried biomass and stirred at 180 rpm for 20 min. Then, algae biomass cells were destroyed in an ultrasonic disperser (Sonorex Super RK 100 H, Bandelin, Germany), a homogenizer, and a mef93.1 degasser (MELFIZ-ultrazvuk, Moscow, Russia). The solvent containing the extracted lipids was centrifuged at 13,000 rpm and filtered under vacuum. The solvent was evaporated to dryness in an ShS 30/250–100-L Top Climcontrol drying cabinet (Mir oborudovaiya, St. Petersburg, Russia) at 60 °C. Total lipids were quantified gravimetrically and expressed as a percentage of dry cell weight.

The antioxidant activity of the isolated lipids in scavenging 2-2-diphenyl-1-picrylhydrazyl (DPPH) free radicals was determined as described previously (Section 4.5).

### 4.9. Determination of Microalgae Carbohydrate (Reducing Sugar) Content

Extraction of carbohydrates was carried out by a slightly modified method of Karemore and Sen [54]. In brief, 50 mg of microalgal biomass was placed in a 100 mL flask and mixed with 50 mL of 2% H_2_SO_4_ (*v*/*v*). The mixture was autoclaved at 121 °C and 15 psi for 30 min. The pH of the mixture in the autoclave was maintained at 7 with 1 M NaOH or H_2_SO_4_. To separate the microalgae into granules, the mixture was centrifuged at 4 °C, 1509× *g* for 10 min. The supernatant was used for carbohydrate analysis.

The method with 3,5-dinitrosalicylic acid was used to determine reducing sugars [55]. When sugars interact with 3,5-dinitrosalicylic acid, the latter is reduced to 3-amino-5-nitrosalicylic acid.

The antioxidant activity of the isolated carbohydrates in scavenging 2-2-diphenyl-1-picrylhydrazyl (DPPH) free radicals was determined as described previously (Section 4.5).

### 4.10. Determination of Antimicrobial Activity of Proteins, Lipids, and Carbohydrates of Microalgae

The diffusion method was used to evaluate the antimicrobial activity of the studied samples in relation to the growth of opportunistic and pathogenic test strains of microorganisms (on a solid nutrient medium).

The following test strains were used as pathogenic and opportunistic bacterial cells: *Proteus vulgaris* ATCC 63, *Escherichia coli* ATCC 25922, *Bacillus subtilis* (B-7918), *Staphylococcus* aureus ATCC 25923, and *Candida albicans* EMTC 34 (Research Institute for Genetics and Selection of Industrial Microorganisms of National Research Center “Kurchatov Institute”, Moscow, Russia).

*E. coli* was cultured on LB medium (FBIS SRCAMB, Obolensk, Russia). The cultures were incubated at pH 7.5–8.0 and 37 °C for 48 h. *S. aureus* was cultured on MPA medium (FBIS SRCAMB, Obolensk, Russia) at pH 6.8–7.2 and 30 °C for 72 h. *P. vulgaris* was cultured on GRM-agar medium (FBIS SRCAMB, Obolensk, Russia) at pH 7.1–7.5 and 37 °C for 24 h. *B. subtilis* was cultivated in Gauze medium (FBIS SRCAMB, Obolensk, Russia) at pH 8.0, temperature 35 °C for 48 h. *C. albicans* was cultured on Sabouraud agar media (FBIS SRCAMB, Obolensk, Russia). The cultures were incubated at 37 °C for 48 h.

To implement the diffusion method (on a solid nutrient medium), the studied test strain was placed on a solid agar nutrient medium and a disk soaked with the test sample was placed on it. A Petri dish with a disc coated with the antibiotic ampicillin was used as a control. The test strains were cultivated for 24.0 ± 0.5 h in an incubator at a constant temperature of 37 °C. The presence and size (in mm) of a transparent zone of the absence of microbial growth around the disk were considered in the results.

### 4.11. Statistical Analysis

The data were subjected to analysis of variance (ANOVA) using Statistica 10.0 (StatSoft Inc., 2007, Tusla, OK, USA). Post hoc analysis (Duncan’s test) was undertaken to identify samples that were significantly different from each other. The equality of the variances of the extracted samples was checked using the Levene test. Differences between means were considered significant when the confidence interval was smaller than 5% (*p* < 0.05).

## 5. Conclusions

The chemical composition of microscopic algae biomass was studied. It was found that microalgae are rich in protein and contain lipids and reducing sugars. To confirm the accuracy of the determination, the protein content was determined using two methods (Kjeldahl and Bradford). The protein content in the microalgae samples *S. intermedius* and *S. obliquus* did not differ significantly when studied by both methods. The lipid content of the microalgae *S. intermedius* samples is eight times lower than the protein content of the *S. intermedius* samples. The lipid content in the *S. obliquus* samples is 8.3 times lower than the protein content in the *S. obliquus* samples. A similar dependence was also observed for reducing sugars contained in the *S. intermedius* and *S. obliquus* samples.

The presence of biological activity (antioxidant) in proteins and lipids extracted from biomass samples of microscopic algae (*S. intermedius*, *S. obliquus*) was demonstrated, laying the foundation for their practical application in the development of biologically active additives for humans and premixes for livestock and poultry. Studies of the antioxidant and antibacterial properties of microalgae are promising, since they are a valuable raw source for obtaining organic substances (amino acids, enzymes, hormones, vitamins, growth substances, antibiotics, and other biologically active compounds) for the food, chemical, and pharmaceutical industries. Furthermore, the use of algae for wastewater treatment, air regeneration, and soil fertility is of great interest, but more research is needed.

## Figures and Tables

**Table 1:** The main species of microalgae and cyanobacteria inhibiting the Baltic Sea.

Division	Family	Species	Source
*Chlorophyta*	Chlorellaceae	*Chlorella* sp.	[6,7]
*Chlorophyta*	Chlorococcaceae	*Chlorococcum* sp.	[8]
*Cyanophyta*	Oscillatoriaceae	*Phormidium* sp.	[6]
*Rhodophyta*	Rhodomelaceae	*Polysiphonia* sp.	[6]
*Cyanophyta*	Oscillatoriaceae	*Lyngbya* sp.	[7]
*Cyanophyta*	Nostocaceae	*Nostoc* sp.	[6,7,8]
*Cyanophyta*	Nostocaceae	*Anabaena* sp.	[7]
*Ochrophyta*	Naviculaceae	*Navicula* sp.	[6,7]
*Ochrophyta*	Bacillariaceae	*Nitzschia* sp.	[6]
*Ochrophyta*	Thalassiophysaceae	*Thalassiophysa* sp.	[6,7,8]
*Ochrophyta*	Catenulaceae	*Amphora* sp.	[8]
*Ochrophyta*	Bacillariaceae	*Bacillaria* sp.	[6,7]
*Cyanophyta*	Synechococcaceae	*Synechococcus* sp.	[6]
*Cyanophyta*	Aphanothecaceae	*Aphanothece* sp.	[7]
*Chlorophyta*	Oocystaceae	*Oocystis* sp.	[6]
*Chlorophyta*	Coccomyxaceae	*Coccomyxa* sp.	[6,7,8]
*Chlorophyta*	Selenastraceae	*Kirchneriella* sp.	[6]
*Ochrophyta*	Amphipleuraceae	*Halamphora* sp.	[6,7]

**Table 2:** Content of proteins, lipids, and reducing sugars in *S. intermedius* and *S. obliquus* microalgae samples (Filinskaya Bay, Baltic Sea) without and after sonication.

Indicator	*S. intermedius*		*S. obliquus*	
	Without Sonification	After Sonification	Without Sonification	After Sonification
Protein (content by Kjeldahl)	51.44 ± 0.7	63.27 ± 0.7	50.07 ± 0.7	60.11 ± 0.7
Protein (content by Bradford)	53.04 ± 0.7	63.46 ± 0.7	50.14 ± 0.7	60.07 ± 0.7
Lipid content	14.13 ± 0.3 *	26.59 ± 0.3	13.73 ± 0.3 *	25.96 ± 0.3 *
Reducing sugar content	15.01 ± 0.3 *	27.13 ± 0.3	14.38 ± 0.3 *	26.72 ± 0.3 *

Values in columns followed by the symbol “*” did not have significant differences (*p* > 0.05) as assessed by post hoc test (Duncan’s test). Data are presented as a mean ± SD (*n* = 3).

**Table 3:** Indicators of antioxidant activity of *S. intermedius* and *S. obliquus* (Filinskaya Bay, Baltic Sea) extract samples.

Sample	DPPH (IC_50_) of Proteins, mg/mL	DPPH (IC_50_) of Lipids, mg/mL	DPPH (IC_50_) of Sugars, mg/mL
Ascorbic acid (control)	0.0084 ± 0.0001		
*S. intermedius*	335.32 ± 10.0 *	0.0026 ± 0.01 *	None
*S. obliquus*	330.16 ± 10.0 *	0.0013 ± 0.01*	None

Values in columns followed by the symbol “*” did not have significant differences (*p* > 0.05) as assessed by post hoc test (Duncan’s test). Data are presented as a mean ± SD (*n* = 3).

**Table 4:** Results of antibacterial activity of proteins, lipids, and sugars obtained from microalgae *S. intermedius* and *S. obliquus* biomass samples (Filinskaya Bay, Baltic Sea).

Components	Studied Sample	Inhibition Zone Diameter, mm				
		*P. vulgaris*	*E. coli*	*B. subtilis*	*S. aureus*	*C. albicans*
Proteins	1	13.0 ± 0.4	0.0 ± 0.0 *	0.0 ± 0.0 *	8.0 ± 0.2	2.0 ± 0.1
	2	6.0 ± 0.2	0.0 ± 0.0 *	0.0 ± 0.0 *	0.0 ± 0.0	3.0 ± 0.1
Lipids	1	4.0 ± 0.3	10.0 ± 0.3	0.0 ± 0.0	4.0 ± 0.1	11.0 ± 0.3 *
	2	3.0 ± 0.3	0.0 ± 0.0	16.0 ± 0.5	12.0 ± 0.3	10.0 ± 0.3 *
Reducing sugars	1	16.0 ± 0.5 *	18.0 ± 0.5	16.0 ± 0.5	19.0 ± 0.6	18.0 ± 0.5 *
	2	16.0 ± 0.5 *	12.0 ± 0.3	14.0 ± 0.4	12.0 ± 0.3	17.0 ± 0.5 *
Control (antibiotic)		26.0 ± 0.8	28.0 ± 0.8	30.0 ± 0.9	22.0 ± 0.6	23.0 ± 0.7

1—*S. intermedius*; 2—*S. obliquus*. Values in columns followed by the symbol “*” did not have significant differences (*p* > 0.05) as assessed by post hoc test (Duncan’s test). Data are presented as a mean ± SD (*n* = 3).

**Table 5:** The content of proteins, lipids, and carbohydrates in the samples of *S. typhimurium* and *S. obliquus* microalgae obtained in various studies.

Values	Microalgae				
	*S. intermedius*	*S. obliquus*	*Scenedesmus* sp.		
	Our Studies		[42,43]	[44]	[45]
Proteins, %	63.27 ± 1.89 *	60.11 ± 1.80 *	44.0	58.7 *	53.2
Lipids, %	26.59 ± 0.79 *	25.96 ± 0.78 *	6.0	21.0 *	12.5
Carbohydrates, %	27.13 ± 0.81 *	26.72 ± 0.79 *	7.0	26.2 *	22.0

Values in rows followed by the symbol “*” did not have significant differences (*p* > 0.05) as assessed by post hoc test (Duncan’s test). Data are presented as a mean ± SD (*n* = 3).

**Table 6:** Values of antioxidant activity of *S. typhimurium* and *S. obliquus* microalgae samples obtained in various studies.

DPPH (IC_50_) of Proteins, mg/mL		DPPH (IC_50_) of Lipids, mg/mL		Sources
*S. intermedius*	*S. obliquus*	*S. intermedius*	*S. obliquus*	
335.32 ± 10.05	330.16 ± 9.90	0.0026 ± 0.0001	0.0013 ± 0.0001 *	Our studies
-	-	-	0.00125 *	[46]

Values in columns followed by the symbol “*” did not have significant differences (*p* > 0.05) as assessed by post hoc test (Duncan’s test). Data are presented as a mean ± SD (*n* = 3).

**Table 7:** Values of antimicrobial activity (diameter of the inhibition zone, mm) in *S. typhimurium* and *S. obliquus* microalgae samples obtained in various studies.

Microalgae	Microorganisms					Sources
	*E. coli*	*C. albicans*	*S. aureus*	*B. subtilis*	*P. vulgaris*	
Lipids						
*S. intermedius*	1.00 ± 0.03	11.00 ± 0.30	4.00 ± 0.11	0.00 ± 0.00	4.00 ± 0.10	Our studies
*S. obliquus*	0.00 ± 0.00	10.00 ± 0.30	12.00 ± 0.36	16.00 ± 0.48	3.00 ± 0.07	
Carbohydrates						
*S. intermedius*	18.00 ± 0.54	18.00 ± 0.54	19.00 ± 0.55	16.00 ± 0.48	16.00 ± 0.48	Our studies
*S. obliquus*	12.00 ± 0.36	17.00 ± 0.51	12.00 ± 0.36	14.00 ± 0.42 *	16.00 ± 0.48	
	-	-	-	12.50 ± 0.37 *	-	[46]
	-	-	-	-	20.00 ± 0.58	[47]

Values in columns followed by the symbol “*” did not have significant differences (*p* > 0.05) as assessed by post hoc test (Duncan’s test). Data presented as a mean ± SD (*n* = 3).

## Data Availability

The data are included in the manuscript.

## Appendix A. *S. intermedius* 18S Ribosomal RNA (18S rDNA) Gene Sequence

CATATCTGTCATTGAGTTGATGGGTATGGACCCTCGTCATTTACTGTGAGCAAAATAGAGTGTTCAAAGCAGGCTTACGCCGTTGAATACATTAGCATGGAATAATAAGATAGGACCTTTGGTCTATTTTGTTGGTTATACTCCGAGGTAATGATTAATAGGGACAGTTGGGGGTATTCGTATTCAATTGTCAGAGGTGAAATTCTTGGATTTATGGAAGACGAACTACTGCGAAAGCATTTACAAGGATGTTTTCATTAATCAAGAACGAAAGTTAGGGGATCGAAGATGATTAGATACCATCGTAGTCTTAACCATAAACTATGCCGACTAGGGATTGGTGGGCGTTTTTTTGACTCCATCAGCACCTTATGAGAAATCAAAGTCTTTGGGTTCCGGGGGGAGTATGGTCGCAAGG CTGAAACTTAAAGAAATTGACGGAAGGGCACCACCAGGAGTGGAGCCTGCGGCTTAATTTGACTCAACACGGGGAAACTTACCAGGTCCAGACATAGTGAGGATTGACAGATTGAGAGCTCTTTCTTGATTCTATGGGTGGTGGTGCATGGCCGTTCTTAGTTGGTGGAGTGATTTGTCTGGTTAATTCCGTTAACGAACGAGACCCCCGCCTGCTAACTAGTCGTCTGAATGCCTAGCATTGAGATGTACTTCTTAGAGGGACTTTCGGTGATTAATCGAAGGAAGTTGGGGGCAATAACAGGTCTGTGATGCCCTTAGATGTCCTGGGCCGCACGCGCGCTACACTGACACACGCAGCGAGTTTTCCTTGTCCGAAAGGTCTGGGTAATCTTGGAAATGTGTGTCGTGATAGGGATAGATTATTGCAATTATTAATCTTGAACGAGGAATTCCTAGTAAATGCGAGTCATCAGCTCGCGTTGATTACGTCCCTGCCCTTTGTACACACCGCCCGTCGCACCTACCGATTGGATGATTCGGTGAAACTTTCGGACCGAAGGTGCGAGACGCTCTCGGGCGACTCGGTCGGGGAAGTTA

## Appendix B. *S. obliquus* 18S rRNA Gene Sequence

AGTTAAAAAGCTCGTAGTTGGATTTCGGGTGGGTTCTAGCGGTCCGCCTATGGTGAGTACTGCTATGGCCTTCCTTTCTGTCGGGGACGGGCTTCTGGGCTTCACTGTCCGGGACTCGGAGTCGACGTGGTTACTTTGAGTAAATTAGAGTGTTCAAAGCAGGCTTACGCCAGAATACTTTAGCATGGAATAACACGATAGGACTCTGGCCTATCTTGTTGGTCTGTAGGACCGGAGTAATGATTAAGAGGGACAGTCGGGGGCATTCGTATTTCATTGTCAGAGGTGAAATTCTTGGATTTATGAAAGACGAACTACTGCGAAAGCATTTGCCAAGGATGTTTTCATTAATCAAGAACGAAAGTTGGGGGCTCGAAGACGATTAGATACCGTCGTAGTCTCAACCATAAACGATGCCGACTAGGGATTGGCGAATGTTTTTTTAATGACTTCGCCAGCACCTTATGAGAAATCAAAGTTTTTGGGTTCCGGGGGGAGTATGGTCGCAAGGCTGAAACTTAAAGGAATTGACGGAAGGGCACCACCAGGCGTGGAGCCTGCGGCTTAATTTGACTCAACACGGGAAAACTTACCAGGTCCAGACATAGTGAGGATTGACAGATTGAGAGCTCTTTCTTGATTCTATGGGTGGTGGTGCATGGCCGTTCTTAGTTGGTGGGTTGCCTTGTCAGGTTGATTCCGGTAACGAACGAGACCTCAGCCTGCTAAATAGTCTCAGTTGCTTTTTGCAGCTGGCTTGACTTTCTTAGA

## References

[1] Koyande A.K., Chew K.W., Rambabu K., Tao Y., Chu D.-T., Show P.-L. (2019). Microalgae: A potential alternative to health supplementation for humans. Food Sci. Hum. Wellness.

[2] Bleakley S., Hayes M. (2017). Algal Proteins: Extraction, Application, and Challenges Concerning Production. Foods.

[3] Makarova E.I., Oturina I.P., Sidyakin A.I. (2009). Applied aspects of the use of microalgae—Inhabitants of aquatic ecosystems. Ecosystems.

[4] Su Y., Song K., Zhang P., Su Y., Cheng J., Chen X. (2017). Progress of microalgae biofuel’s commercialization. Renew. Sustain. Energy Rev..

[5] Amorim M.L., Soares J., Coimbra J.S.D.R., Leite M.D.O., Albino L.F.T., Martins M.A. (2020). Microalgae proteins: Production, separation, isolation, quantification, and application in food and feed. Crit. Rev. Food Sci. Nutr..

[6] Wiśniewska K., Śliwińska-Wilczewska S., Lewandowska A., Konik M. (2021). The Effect of Abiotic Factors on Abundance and Photosynthetic Performance of Airborne Cyanobacteria and Microalgae Isolated from the Southern Baltic Sea Region. Cells.

[7] Lewandowska A.U., Śliwińska-Wilczewska S., Woźniczka D. (2017). Identification of cyanobacteria and microalgae in aerosols of various sizes in the air over the Southern Baltic Sea. Mar. Pollut. Bull..

[8] Sarayloo E., Simsek S., Unlu Y.S., Cevahir G., Erkey C., Kavakli I.H. (2018). Enhancement of the lipid productivity and fatty acid methyl ester profile of Chlorella vulgaris by two rounds of mutagenesis. Bioresour. Technol..

[9] Soto-Sierra L., Dixon C.K., Wilken L.R. (2017). Enzymatic cell disruption of the microalgae Chlamydomonas reinhardtii for lipid and protein extraction. Algal Res..

[10] Benemann J. (2013). Microalgae for biofuels and animal feeds. Energies.

[11] Ba F., Ursu A.V., Laroche C., Djelveh G. (2016). Haematococcus pluvialis soluble proteins: Extraction, characterization, concentration/fractionation and emulsifying properties. Bioresour. Technol..

[12] Ojha K.S., Alvarez C., Kumar P., O’Donnell C., Tiwari B.K. (2016). Effect of enzymatic hydrolysis on the production of free amino acids from boarfish (*Capros aper*) using second order polynomial regression models. LWT-Food Sci. Technol..

[13] Klis F.M., de Jong M., Brul S., de Groot P.W.J. (2007). Extraction of cell surface-associated proteins from living yeast cells. Yeast.

[14] Jubeau S., Marchal L., Pruvost J., Jaouen P., Legrand J., Fleurence J. (2012). High pressure disruption: A two-step treatment for selective extraction of intracellular components from the microalga Porphyridium cruentum. J. Appl. Phycol..

[15] Batista A.P., Gouveia L., Bandarra N.M., Franco J.M., Raymundo A. (2013). Comparison of microalgal biomass profiles as novel functional ingredient for food products. Algal Res..

[16] Borowitzka M.A. (2013). High-value products from microalgae—their development and commercialisation. J. Appl. Phycol..

[17] Suganya T., Varman M., Masjuki H.H., Renganathan S. (2016). Macroalgae and microalgae as a potential source for commercial applications along with biofuels production: A biorefinery approach. Renew. Sustain. Energy Rev..

[18] Bai M.-D., Chen C.-Y., Lu W.-C., Wan H.-P., Ho S.-H., Chang J.-S. (2015). Enhancing the oil extraction efficiency of Chlorella vulgaris with cell-disruptive pretreatment using active extracellular substances from Bacillus thuringiensis ITRI-G1. Biochem. Eng. J..

[19] Scharff C., Domurath N., Wensch-Dorendorf M., Schröder F.-G. (2017). Effect of different photoperiods on the biochemical profile of the green algae *C. vulgaris* and *S. obliquus*. Acta Hortic..

[20] Sakarika M., Kornaros M. (2016). Effect of pH on growth and lipid accumulation kinetics of the microalga Chlorella vulgaris grown heterotrophically under sulfur limitation. Bioresour. Technol..

[21] Islam M.A., Brown R.J., O’Hara I., Kent M., Heimann K. (2014). Effect of temperature and moisture on high pressure lipid/oil extraction from microalgae. Energy Convers. Manag..

[22] Li Z., Li Y., Zhang X., Tan T. (2015). Lipid extraction from non-broken and high water content microalgae Chlorella spp. by three-phase partitioning. Algal Res..

[23] Karyakin D.O., Maltsevskaya N.V., Novicheva M.V., Kulabukhov V.Y. (2017). Optimization of the culture medium for *Scenedesmus* sp. Int. Res. J..

[24] Rocha R.P., Machado M., Vaz M.G.M.V., Vinson C.C., Leite M., Richard R., Mendes L.B.B., Araujo W.L., Caldana C., Martins M.A. (2017). Exploring the metabolic and physiological diversity of native microalgal strains (Chlorophyta) isolated from tropical freshwater reservoirs. Algal Res..

[25] Soares L.D.S., de Faria J.T., Amorim M.L., de Araújo J.M., Minim L.A., Coimbra J.S.D.R., Teixeira A.V.N.D.C., de Oliveira E.B. (2017). Rheological and Physicochemical Studies on Emulsions Formulated with Chitosan Previously Dispersed in Aqueous Solutions of Lactic Acid. Food Biophys..

[26] Choi S.P., Nguyen M.-T., Sim S.J. (2010). Enzymatic pretreatment of Chlamydomonas reinhardtii biomass for ethanol production. Bioresour. Technol..

[27] Moreno F.J. (2014). Fractionation of Food Bioactive Oligosaccharides Food Oligosaccharides: Production, Analysis and Bioactivity.

[28] DuBois M., Gilles K.A., Hamilton J.K., Rebers P.A., Smith F. (1956). Colorimetric method for determination of sugars and related substances. Anal. Chem..

[29] Parniakov O., Roselló-Soto E., Barba F.J., Grimi N., Lebovka N., Vorobiev E. (2015). New approaches for the effective valorization of papaya seeds: Extraction of proteins, phenolic compounds, carbohydrates, and isothiocyanates assisted by pulsed electric energy. Food Res. Int..

[30] Montanes F., Corzo N., Olano A., Reglero G., Ibanez E., Fornari T. (2008). Selective fractionation of carbohydrate complex mixtures by supercritical extraction with CO2 and different co-solvents. J. Supercrit. Fluids.

[31] El-Sheekh M., Fathy A.A. (2009). Variation of Some Nutritional Constituents and Fatty Acid Profiles of Chlorella vulgaris Beijerinck Grown under Auto and Heterotrophic Conditions. Int. J. Bot..

[32] Liu J., Yuan C., Hu G., Li F. (2012). Effects of Light Intensity on the Growth and Lipid Accumulation of Microalga *Scenedesmus* sp. 11-1 Under Nitrogen Limitation. Appl. Biochem. Biotechnol..

[33] Sforza E., Simionato D., Giacometti G.M., Bertucco A., Morosinotto T. (2012). Adjusted Light and Dark Cycles Can Optimize Photosynthetic Efficiency in Algae Growing in Photobioreactors. PLoS ONE.

[34] Simionato D., Sforza E., Corteggiani Carpinelli E., Bertucco A., Giacometti G.M., Morosinotto T. (2011). Molecular and phylogenetic identification of an oil-producing strain of *Nannochloropsis oceanica* (Eustigmatophyceae) isolated from the southwestern Atlantic coast (Argentina). Bioresour. Technol..

[35] Cheirsilp B., Torpee S. (2012). Enhanced growth and lipid production of microalgae under mixotrophic culture condition: Effect of light intensity, glucose concentration and fed-batch cultivation. Bioresour. Technol..

[36] Solovchenko A., Khozin-Goldberg I., Didicohen S., Cohen Z., Merzlyak M.N. (2007). Effects of light intensity and nitrogen starvation on growth, total fatty acids and arachidonic acid in the green microalga Parietochloris incisa. J. Appl. Phycol..

[37] Ruangsomboon S. (2012). Effect of light, nutrient, cultivation time and salinity on lipid production of newly isolated strain of the green microalga, Botryococcus braunii KMITL 2. Bioresour. Technol..

[38] Breuer G., Lamers P.P., Martens D.E., Draaisma R.B., Wijffels R.H. (2013). Effect of light intensity, pH, and temperature on triacylglycerol (TAG) accumulation induced by nitrogen starvation in Scenedesmus obliquus. Bioresour. Technol..

[39] Grobbelaar J.U., Nedbal L., Tichý V. (1996). Influence of high frequency light/dark fluctuations on photosynthetic characteristics of microalgae photoacclimated to different light intensities and implications for mass algal cultivation. J. Appl. Phycol..

[40] Xue S., Zhang Q., Wu X., Yan C., Cong W. (2013). A novel photobioreactor structure using optical fibers as inner light source to fulfill flashing light effects of microalgae. Bioresour. Technol..

[41] Masojídek J., Torzillo G., Koblížek M., Kopecký J., Bernardini P., Sacchi A., Komenda J. (1999). Photoadaptation of two members of the Chlorophyta (Scenedesmus and Chlorella) in laboratory and outdoor cultures: Changes in chlorophyll fluorescence quenching and the xanthophyll cycle. Planta.

[42] Starokadomsky D., Titenko A., Kamarali A., Kuts V., Maloshtan S., Barkholenko V., Kashuba O., Reshetnik M., Starokadomskaya A., Diamant V. (2021). Review of scientific works on technologies of extraction of biocomponents from plant raw material. supercritical CO_2_-extraction is an effective new method for solving the global problem of utilization and quality of plant and organic raw materials. Globe Eng. Sci..

[43] Vieira B.B., Soares J., Amorim M.L., Bittencourt P.V.Q., Superbi R.D.C., de Oliveira E.B., Coimbra J.S.D.R., Martins M.A. (2021). Optimized extraction of neutral carbohydrates, crude lipids and photosynthetic pigments from the wet biomass of the microalga Scenedesmus obliquus BR003. Sep. Purif. Technol..

[44] Masuko T., Minami A., Iwasaki N., Majima T., Nishimura S.-I., Lee Y.C. (2005). Carbohydrate analysis by a phenol–sulfuric acid method in microplate format. Anal. Biochem..

[45] Patnaik R., Singh N.K., Bagchi S.K., Rao P.S., Mallick N. (2019). Utilization of Scenedesmus obliquus Protein as a Replacement of the Commercially Available Fish Meal Under an Algal Refinery Approach. Front. Microbiol..

[46] Bradford M.M. (1976). A rapid and sensitive method for the quantitation of microgram quantities of protein utilizing the principle of protein-dye binding. Anal. Biochem..

[47] Rumin J., Nicolau E., Junior R.G.D.O., Fuentes-Grünewald C., Flynn K.J., Picot L. (2020). A Bibliometric Analysis of Microalgae Research in the World, Europe, and the European Atlantic Area. Mar. Drugs.

[48] Zaharieva M.M., Zheleva-Dimitrova D., Rusinova-Videva S., Ilieva Y., Brachkova A., Balabanova V., Gevrenova R., Kim T.C., Kaleva M., Georgieva A. (2022). Antimicrobial and Antioxidant Potential of *Scenedesmus obliquus* Microalgae in the Context of Integral Biorefinery Concept. Molecules.

[49] Makarova N.V., Zyuzina A.V. (2011). Investigation of antioxidant activity by the DPPH method of semi-finished products of juice production. Tech. Technol. Food Prod..

[50] Marrez D.A., Naguib M.M., Sultan Y.Y., Higazy A.M. (2019). Antimicrobial and anticancer activities of Scenedesmus obliquus metabolites. Heliyon.

[51] Raut G., Kamat S., RaviKumar A. (2019). Trends in production and fuel properties of biodiesel from heterotrophic microbes. Advances in Biological Science Research.

[52] Montegiove N., Pellegrino R., Emiliani C., Pellegrino A., Leonardi L. (2021). An Alternative Approach to Evaluate the Quality of Protein-Based Raw Materials for Dry Pet Food. Animals.

[53] Folch J., Lees M., Stanley G.H.S. (1957). A simple method for the isolation and purification of total lipides from animal tissues. J. Biol. Chem..

[54] Bolobanshchikova G.N., Rogozin Y.D. (2017). Diatoms of bottom sediments of nearby lakes of Khakassia. Bulletin of the Buryat State University. Chem. Phys..

[55] Barbarino E., Lourenço S.O. (2006). An evaluation of methods for extraction and quantification of protein from marine macro- and microalgae. J. Appl. Phycol..

